# Social media text analytics of Malayalam–English code-mixed using deep learning

**DOI:** 10.1186/s40537-022-00594-3

**Published:** 2022-04-26

**Authors:** S. Thara, Prabaharan Poornachandran

**Affiliations:** Department of Computer Science and Engineering, Amrita Vishwa Vidyapeetham, Amritapuri, India

**Keywords:** Deep neural network, Dravidian languages, Natural Language Processing, Multilingual language

## Abstract

Zigzag conversational patterns of contents in social media are often perceived as noisy or informal text. Unrestricted usage of vocabulary in social media communications complicates the processing of code-mixed text. This paper accentuates two major aspects of code mixed text: Offensive Language Identification and Sentiment Analysis for Malayalam–English code-mixed data set. The proffered framework addresses 3 key points apropos these tasks—dependencies among features created by embedding methods (Word2Vec and FastText), comparative analysis of deep learning algorithms (uni-/bi-directional models, hybrid models, and transformer approaches), relevance of selective translation and transliteration and hyper-parameter optimization—which ensued in F1-Scores (model’s accuracy) of 0.76 for Forum for Information Retrieval Evaluation (FIRE) 2020 and 0.99 for European Chapter of the Association for Computational Linguistics (EACL) 2021 data sets. A detailed error analysis was also done to give meaningful insights. The submitted strategy turned in the best results among the benchmarked models dealing with Malayalam–English code-mixed messages and it serves as an important step towards societal good.

## Introduction

Social networking has been proliferating worldwide, over the last decade, concurrent with remarkable advancements in communication technologies. Research in Indian languages has evinced keen interest [1]. In India, social media enthusiasts originate from regions of diverse languages and multi-cultural backgrounds [2]. Indian civilization was endowed with an enriched linguistic heritage. Britain’s 200-year colonization enabled India to become the second largest English-speaking population.^1^ Malayalam, a Dravidian^2^ language mainly spoken in the southern parts of India, is the official language of the Union Territories—Lakshadweep and Pondicherry, and the state Kerala, India. A deeply agglutinative language, the global Malayalam-speaking population is nearly 38 million [3]. The alphabets in Malayalam are constituted of the Vatteluttu^3^ alpha-syllabic scripts that belong to a family of the Abugida^4^ writing system. Social media enthusiasts often adopt the Roman script as language scripts due to its ease of input. Hence, majority of the data content in social media, available for under-resourced languages, is code-mixed [4].

The prime objective of this research has been to identify sentiment polarity and repulsive content in Malayalam–English code-mixed blogs in social media. This study highlights the latent interspersed offensive language and sentiment polarity content in the Dravidian languages, on social media. Also, the research is evaluated by various techniques and outperforms well with the published results [5]. Sentiment analysis (SA) has been an active area of research since the advent of the current millennium. Code-mixed texts in social media have spurred the demand for SA [6–8]. SA entails the identification of subjective opinions or responses on a given topic, product or service in e-commerce. Progressive evolutions in communications and networking technologies have motivated consumers to share their personal opinions and critiques of retail products and services in real time.

The ensuing fallout of aggressive, harmful posts on social websites is undeniable. Nowadays, people openly voice their disdain towards a government policy or specific individuals, by posting abusive critical bulletins. The deluge of derisive fictitious messages must be detected and suppressed in any communicative forum. Such falsified posts profusely hurt people’s sentiments, causing mental trauma, and distress [9–11]. Unrestrained proclivity towards dissemination of fake news and derogatory contents calls for their automatic detection and proscription from media platforms [12]. To some extent, denigratory communications have been forbidden in the English language. Nevertheless, prevention of resentful Indian language blogs, in the code-mixed domain, is in early infancy stages [13].

The exigency of this problem is critical in societal domains such as health care, politics, e-retail, and movie review. In the prevailing digital era, people prefer the social media for news updates, and interaction with friends. Media blogs are replete with natural language content. Governmental agencies [14] have enacted strict laws to deal with proliferation of hateful text through social media and mobile apps. Misinformation on the prevention and cure of the COVID-19 [15] pandemic have serious repercussions on public health, leading to avoidable mental trauma, and distress [16]. Besides, as major business, entertainment, and political activities have been confined to online settings, the deceptive corpus is wildly rampant. Politicians exchange views on latest partisan developments, inviting the citizens to comment, share ideas apropos their political agenda. Public penchant for online entertainment and e-commerce has transformed retail business to movies online and recurrent impulsive shopping. Principal schemes for SA and offensive language identification (OLI) emanated from the computational linguistics domain [17, 18], exploiting the syntactic structure and pragmatic features of code-mixed semantics. Machine learning [19] flourished via the n-gram (sequence of written symbols of length *n*, where *n* can be 1, 2 or 3) word and char features. Character n-gram models incorporate information about the internal structure of the word in terms of character n-gram embedding. Term frequency–Inverse document frequency (TF-IDF) [20] was applied as feature extraction method for the SA and OLI tasks; other measures have leaned on deep learning [21, 22] and ensemble approaches [23, 24].

A major research gap, pinpointing a limited data set [3, 25, 26] called for the creation of a code-mixed corpus due to the unavailability of an openly accessible gold standard data set. Inadequate multi-lingual code-mixed data for fine-tuning pretrained models is another challenge. These under-resourced morphologically rich languages (Malayalam, Tamil, Telugu, Kannada) lack pre-trained models to process low-resource languages. Few state-of-the-art (SOTA) models were adopted to address the research problem of identification of sentiment polarity and offensive content in Malayalam–English code-mixed which yielded F1-scores (measure of a model’s accuracy) of 0.76 and 0.99, respectively, for FIRE 2020 and EACL 2021 data sets of social media text analytics. These were the best results scored for both the data set [27]. The novelty lies in the selective translation and transliteration stages [28], concurrent with optimization of hyper-parameters (learning rate, epochs, optimizers etc.) and up-sampling strategy. Selective translation and transliteration are prime concepts wherein Romanized sentences are converted to their native language where the semantic meaning is preserved. A systematic comparison of deep learning models—Convolution Neural Networks (CNN), Long Short Term Memory (LSTM), Gated Recurrent Unit (GRU), Bidirectional LSTM (BiLSTM), and Bidirectional GRU (BiGRU) returned good results, as a testimony to the propriety of preprocessing of inputs to the proposed model. This research was undertaken for the societal good as unwarranted pejorative posts exacerbate traumatic impairment of a person’s mental health.

Challenges encountered in the run-up to both the tasks were:

(i) short-length messages.
(ii) informal words like plz/pls—please; lvl-level.
(iii) abbreviations like cr—crore; fdfs—first day first show, bgm— back-groundmusic.
(iv) spelling variations like w8/wt – wait; wtng/w8ing/wting,—waiting; avg—average.
(v) emoticons.
(vi) time stamps, various formats are used to mention time, like 3:02, 3 min 3, 3.03 min.
(vii) repetition of characters in words for example coooool, sooooooooperb/supeerb, fansss, maaaaaaaasss.
(viii) multiple ways to represent numbers for example 3.7k, 3700, one lac, one lakh, 2 Million, 2 M.

### Key contributions of this paper

1. A fine-grained analysis of sentiment and offensive content identification, with several deep learning algorithms for the Malayalam–English code-mixed data set.
2. Highlight the propriety of selective translation and transliteration in code-mixed data set.
3. Achievement of benchmark results—overall 2% increase in the F1-score (measure of a model’s accuracy on a data set).
4. Extensive experimental studies with detailed error analysis which stimulate self-directed research.

This paper is organized as follows—section "Related works" presents an overview of the related works. Section "Proposed approach" describes the proposed approach which includes data description, data preprocessing, feature extraction methods, and deep learning approaches. Section "Experimental Setup" discusses the experimental setup for verifications of the proffered design, which include the best hyper-parameter configuration. Section "Results and discussions" discusses results and the inferences from this study. Section "Limitations" gives glimpses of limitations of proposed method. Section "Conclusion" concludes the paper with closing remarks. Section "Future work" draws attention to future research in this area.

## Related works

Researchers have relied on various methods for the complex task of discernment of sentimental offensive language in the code-mixed domain. Initially researchers developed an engineering approach for SA [29], by leveraging a handful of metadata, lexical and sentiment features, to design a model. Several works [30] proposed a combination of Naive Bayes and SVM (NBSVM), for classification of code-mixed data sets. The preprocessing stages included tokenization, hashtag segmentation, URL removals, and lower-casing of sentences. F1-scores of 0.72, 0.65, 0.76 were obtained for negative, neutral, and positive classes, respectively. A unique Enhanced Language Representation with Informative Entities (ERNIE) model [31], was proposed by Liu et al. [32] and applied to code-mixed data sets of Hindi and English. An adversarial training was applied while training, along with XLM-RoBERTa model (XLM-R), for a multilingual model; it achieved F1-scores of 0.799, 0.769 and 0.689 for positive, negative, and neutral classes, respectively. Table 1 summarises few articles. It is divided into Data set, Methodology, Limitations and results.

**Table 1:** Recent references in a nutshell

Data set	Methodology	Limitations	Results
Tamil and Malay alam [33]	A sub-word level to-kenizer, a text rep resentation layer, and a transformer model for classification	Could not identify sarcasm used in negative comments	F1-score of 0.58 and 0.66 average-F1 for Tamil and Malay- alam code-mixed datasets
Hindi-English and Spanish–English data sets [34]	Ensemble of self-attention-based Long Short Term Mem- ory (LSTM), and convolutional neural network (CNN)	Data imbalances are not handled	F1-score of 0.707 and 0.725 respectively
Hindi-English [35]	LSTM network, with character-level embedding and a FastText embedding	Issue in short sentences which has unclear semantic structure	F1-score of 0.679
English and Spanish [36]	Multilingual XLM-R	Computationally intensive and failed to see the patterns in the results	F1-score of 0.537
Hinglish [37]	One-Dimensional (1-D) convolution and 1-D max-pooling, self-attention mech- anisms, and finally, the dense layer	Lack of good pretrained models and hyper-parameter optimization	F1-score of 0.684

Research in OLI [38] has been also facilitated by making the corpus available. Chakravarthi et al. [12] used TF-IDF vectors, along with character level n-grams, towards feature engineering process. Character n-gram models incorporate the internal structure of the word in terms of character n-gram embedding. The four main developed models were—LSTM, LR, XGBoost and attention networks. Traditional ML classifiers with these features produced good F1-score of 0.78, on par with deep learning models [39].

The background studies of SA and OLI in code-mixed corpora address the diverse paths ranging from feature engineering to task modeling. The relevance of this probe was attested by the linguistically diverse code-mixed corpora. Multi-lingual languages are semantically complex, bereft of sophisticated models in the Dravidian code-mixed domain, for the management of SA and OLD [27, 40]. As shown in the above studies, no relevant probe was conducted with selective translation and transliteration incorporating hyper-parameter optimization. This paper undertook a pioneering attempt, for assessment of the viability of selective transliteration and translation preprocessed comments [41], factoring in class imbalances, together with hyper-parameter optimization which augmented the proffered approach’s weighted accuracy (F1-score). Hyper-parameter optimization was shown to be propitious for the final step of tag prediction. An extensive comparative study of several deep learning approaches was conducted; despite data scarcity for code-mixed corpus, the proposed approach attained the best score of 0.76 for the FIRE 2020 and 0.99 for the EACL 2021 data sets.

## Proposed approach

**Fig. 1:**
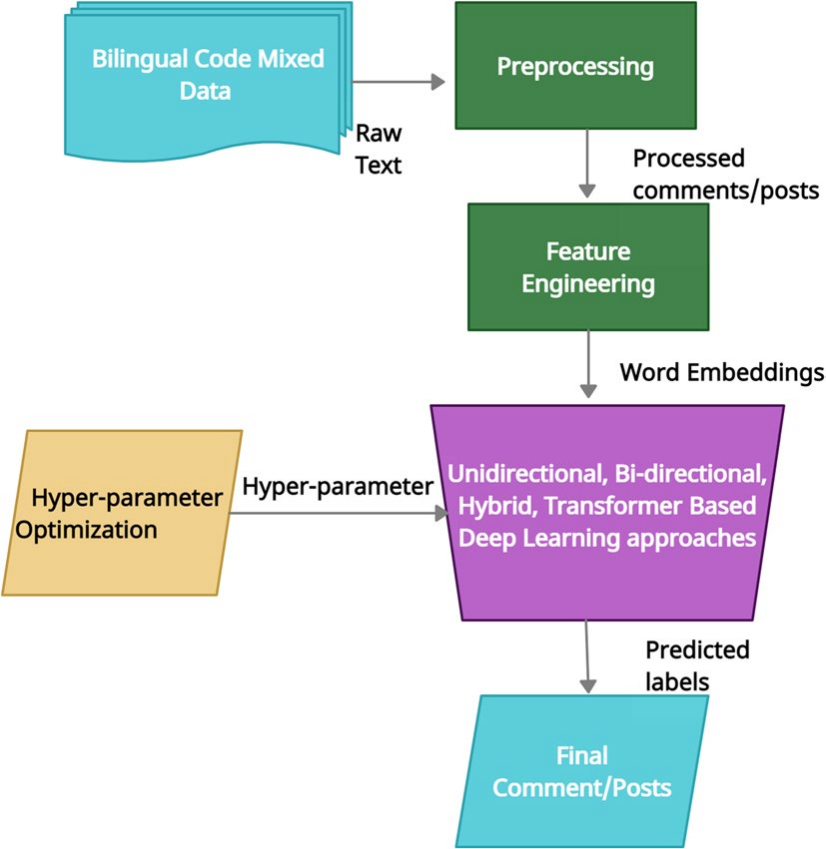
Flow diagram of the proposed work

This section covers a brief on the data set, and feature extraction methods—Word2Vec [42] and FastText [43]. Discussions of SOTA deep learning approaches follows next, inclusive of the requisite hyper-parameters. The section concludes with sentiment and offensive comments/posts prediction. Figure 1 unveils an overview of the proposed methodology, where the different stages are the bilingual code-mixed data set, requisite data preprocessing steps, feature engineering techniques (Word2Vec and FastText), optimized hyper-parameter values for selecting deep learning approaches for modeling and finally prediction.

### Data set description

For the experimental study, the data sets for SA and OLI tasks were retrieved from organizers of the FIRE 2020^5^ and EACL 2021,^6^ who conducted pioneering structured shared tasks in Malayalam–English code-mixed data sets. Hence, the corresponding data sets can be considered as the standard/benchmark data sets for their respective tasks. The comments/posts of the SA, OLI tasks contain more than one sentence, but the average length of sentences of the code-mixed data sets is 1 for both the tasks. Each comment/post is annotated with its corresponding class label. All the tasks can be considered as a fine-grained analysis, as the comments are scrutinized at a finer level.

The code-mixed data set for SA is classified into 5 classes: Positive, Negative, Unknown state, Mixed feelings, and not-Malayalam. The shared task for SA is constituted of 4851 code-mixed social media comments/posts in the training set, 540 comments in the validation data set and 1348 comments in the test data set. The OLD data set is classified into 5 categories: Not offensive (NF), Offensive Targeted Insult Individual (OTII), not Malayalam (NM), Offensive Targeted Insult Group (OTIG) and Offensive Untargetede (OUT). The shared task for offensive language detection (OLD) is comprised of 16,010 code-mixed social media comments/posts in the training set, 1999 comments/posts in the validation set and 2001 comments in the test data set. Since both the SA and OLD tasks involve a fine-grained approach, the availability of training data per class, is minimal. This gives rise to class imbalance problems (non-uniform distribution of classes in the data set), for the two code-mixed data sets, depicting real-world scenarios (Table 2 presents statistics for the SA stats, Table 3 for statistics of the OLD data set). Random up-sampling technique was used to address the class imbalance problems for both the tasks. Hence, minority classes were sampled repeatedly, so that all classes in each of the data sets have an equal number of samples. Percentage distribution of each class in all the three sets (training, validation and test) are shown separately.

**Table 2:** SA data statistics [12]

Class	Train	Valid	Test
Positive	2,022 (41.68%)	224 (41.48%)	565 (41.91%)
Unknown state	1,344 (27.70%)	161 (29.81%)	398 (29.52%)
Not-malayalam	647 (13.33%)	60 (11.11%)	177 (13.13%)
Negative	549 (11.31%)	51 (9.44%)	138 (10.23%)
Mixed feeling	289 (5.95%)	44 (8.14%)	70 (5.19%)
Total	4851	540	1348

**Table 3:** Offensive language detection—OLD data statistics [44]

Class	Train	Valid	Test
Not offensive	14,153 (88.4%)	1779 (88.99%)	1770 (88.5%)
Not-Malayalam	1287 (8.03%)	163 (8.15%)	161 (8.04%)
Offensive Targeted Insult Individual	239 (1.49%)	24 (1.20%)	29 (1.44%)
Offensive Untargeted	191 (1.19%)	20 (1.00%)	24 (1.19%)
Offensive Targeted Insult Group	140 (0.87%)	13 (0.65%)	17 (0.84%)
Total	16 010	1999	2001

### Data set preprocessing

**Fig. 2:**
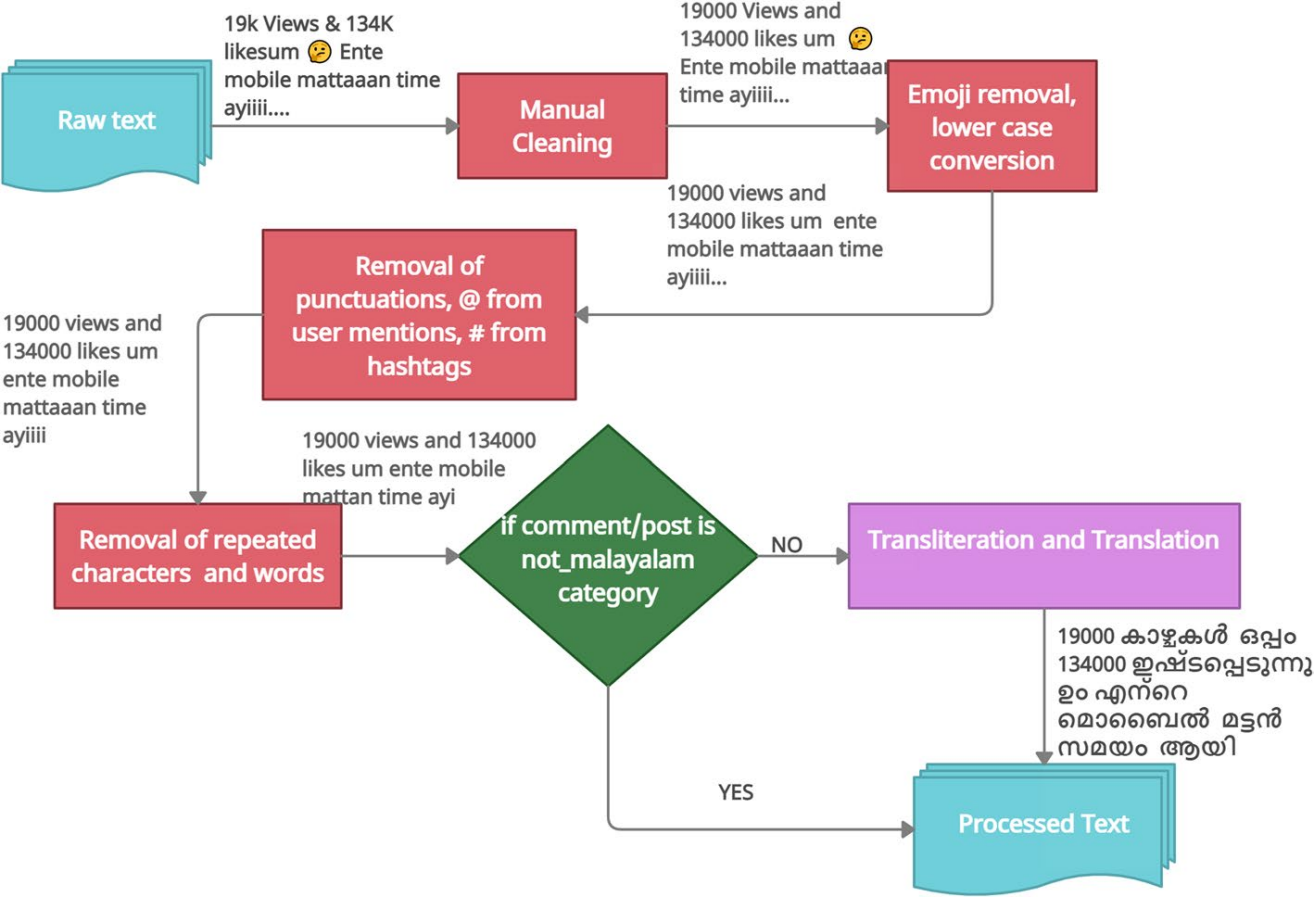
Various stages of preprocessing


Sentimental and Offensive language code-mixed FIRE 2020 and EACL 2021 data sets are constituted of comments/posts from YouTube channel; spelling mistakes and commonly used internet jargon are widely observed within both the data sets. Preprocessing in Malayalam–English code-mixed data set is a challenging task; hence, both the data sets went through the sequence of step shown in Fig. 2. The pseudo code for basic preprocessing is shown in Algorithm 1.

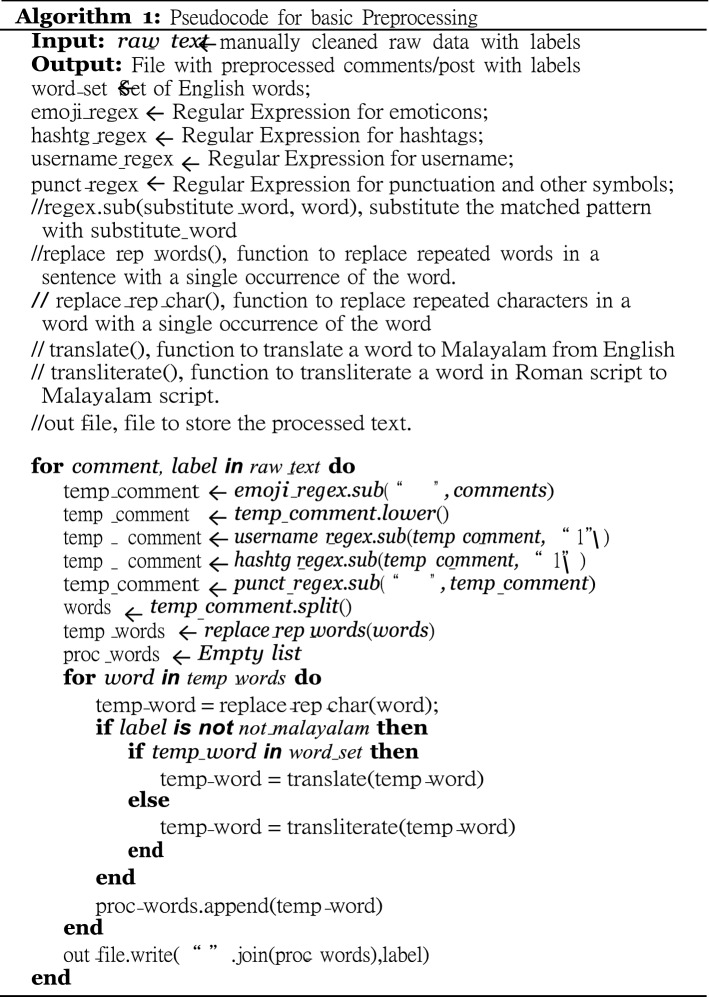


### Selective translation and transliteration

To convert code-mixed text into a native script, we cannot rely on neural translation systems, particularly in tweets where users are prone to write informally, using multiple languages. Besides, translation of Romanized non-English language words into a particular language does not make any sense. In many cases, proper translation of words from English to a non-English language would not be available. We propose selective transliteration and translation of the text as a solution to this problem. In effect, the process of conversion of Romanized text (for example, Manglish) is to transliterate the words in the native Malayalam language in text into Malayalam and translate the English words in the text into Malayalam selectively. This separation of English words from native language words is carried out using a big corpus of English words from NLTK^7^ corpus. The idea of this selective conversion is based on the observation that in the Romanized native language, users tend to use English words only when the word-meaning is better conveyed with the English word, or when the corresponding native language word is not commonly used in regular conversations. For example, Malayalam-users prefer the word “movie” over much its corresponding Malayalam word. During the basic phase, the raw text of the not-Malayalam category are left unchanged; otherwise, the comments/posts are transliterated and translated. The pseudo code for transliteration and translation is shown in Algorithm 2. Google API was used for translation.^8^

### Feature extraction method

Humans can intuitively deal with natural language or text data. A computer’s inability to handle such data call for the numerical representation of texts. A method is needed that can capture the syntactic and semantic relationships among words, along with a clear understanding of the contexts in which they are used in. Such methods are called Word embeddings [45], wherein each word is mapped to an N-dimensional real vector. One of the early methods to form vector representations of a word is called one-hot encoded vector, where a “1” is assigned for the index position of the word and “0” placed elsewhere. Word2Vec and FastText are other word embedding models discussed in the paper.

#### Word2Vec

The Word2Vec embedding method marshals algorithmic schemes such as Continuous Bag of Words (CBOW) [42] and Skip-gram [42] for the derivation of real-valued vectors. These models create embeddings based on the co-occurrence of words; for e.g., ‘She walked by the riverbank, and went to the bank to deposit money’; if we take the word “bank” and the corpus contains more information about riverbanks rather than the financial institution, then the embeddings of the bank will be inclined towards river and stream, instead of finance, lender, etc. Another key limitation is that Word2Vec does not keep track of the position of a word in the sentence i.e., word ordering information is not preserved. Word2Vec ignores the internal structure of the word.
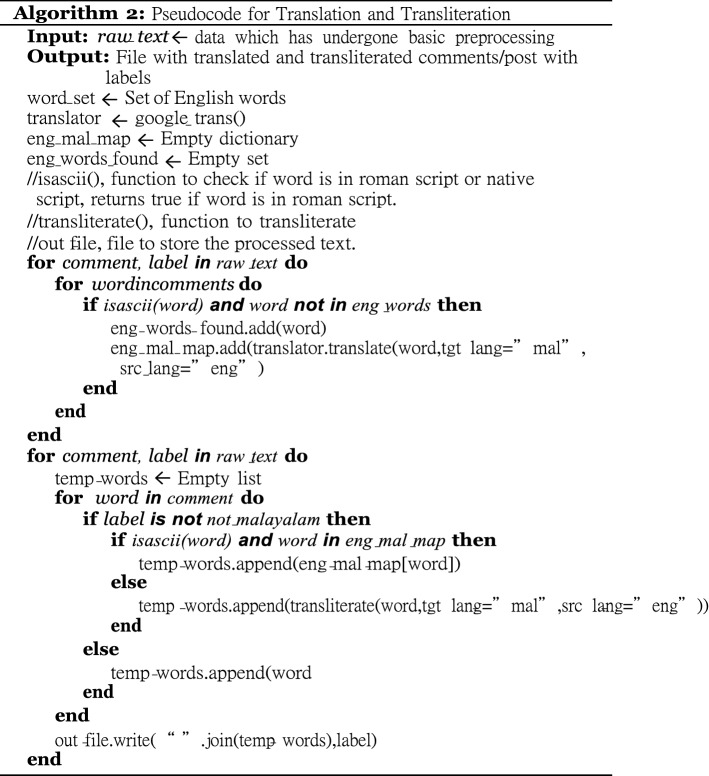


#### FastText

Words that belongs to morphologically rich languages are better handled by character level information (character n-grams). Each word is represented as a bag of character n-grams. We can resolve Out of Vocabulary (OOV) words using this method. FastText is an algorithm which follows character n-gram based model. For each word, the algorithm considers character n-grams for example unigram, bigrams, trigram, five grams etc. We can find shorter and longer n-grams. Shorter n-grams helps to identify the structure of a word; longer n-grams are good to capture its semantic information. Gensim^9^ implementation of Word2Vec and FastText was used to custom train the word embedding model with the two code-mixed data sets. Word2Vec and FastText were trained separately on these two data sets. The learned embeddings were saved in a text file. The Python NumPy^10^ package was used to convert the word embeddings in the text file to a matrix; this word embedding matrix was then used to initialize the weights of Keras Embedding Layer;^11^ the Embedding Layer’s trainable attribute was set to false so that the word embeddings remain constant throughout the training phase.

### Deep learning approaches

In this section the major focus is on the proposed deep learning models used for the prediction of sentiments and offensive content in the Malayalam–English code-mixed domain.

#### CNN

**Fig. 3:**
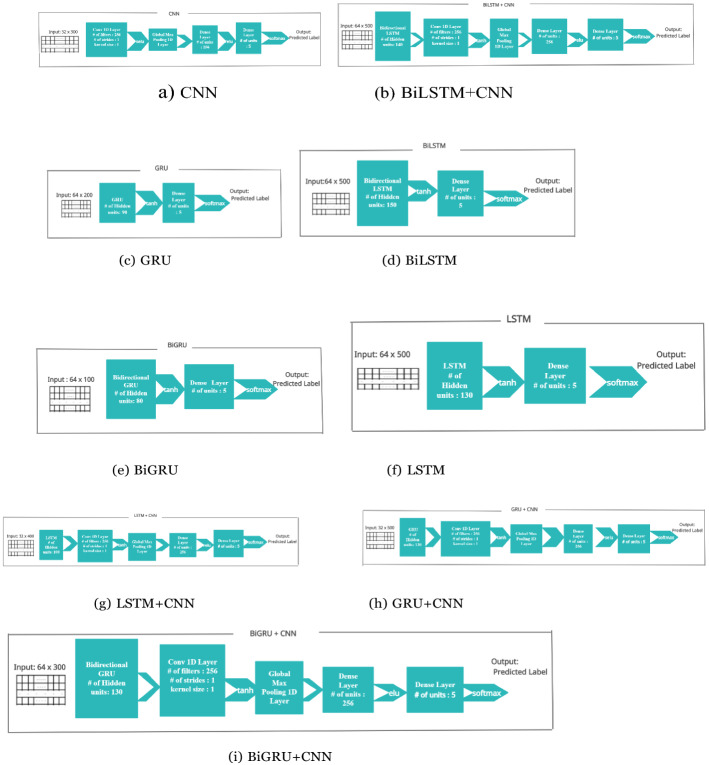
Layer configuration and parameter settings of various deep learning models used in the proposed approach

For this paper’s study, 1D-CNN was used, which is fed with spatially dropped word embeddings; 1-D CNN was used with a kernel size of 1, and 256 filters. The output from the 1-D CNN is down sampled, using 1-D global max pooling. The condensed representation after pooling, is made to pass through two feedforward neural networks, with a dropout layer in between, to avoid over-fitting. The final feed-forward layer acts as a classification layer. Illustrations in given in Fig. 3a.

#### LSTM

In this paper’s investigation, word embeddings were made to pass through a 1D Spatial Dropout layer, which is used to expand the dropout value over the entire feature map. After dropout, the word embeddings are then fed into the LSTM. We have considered the output from the last time step of the LSTM as encoded representation of the input string, followed by, passing the LSTM output through a dropout layer, and then, fed into a feed-forward neural network. The feed forward neural network acts as the classification layer. Pictorial representation is given in Fig. 3f.

#### GRU

In order to reduce overfitting, the word embeddings are fed into the GRU, after a spatial dropout. The last hidden state of an input sentence is made to pass through a dropout layer, followed by the classification layer as shown in Fig. 3c.

#### BiLSTM

BiLSTM yields a more powerful and richer representation of the input sentence, compared to unidirectional LSTM. As shown in Fig. 3d using a single LSTM after spatial dropout, the input sentence is fed to the LSTM, in both the forward and backward directions. The hidden states of the last time step, from both the directions of the input, are concatenated and fed into a dropout layer, which is followed by a feed forward layer that acts as the classification layer.

#### BiGRU

In Fig. 3e the input sentence is made to pass through a spatial dropout layer, before being fed into the BiGRU where the GRU is fed with the input sentence in both the directions. After dropout, the hidden state from the last time step is conveyed to a classification layer.

#### BiLSTM + CNN and LSTM + CNN

BiLSTM + CNN/LSTM + CNN were inspired from the encoder-decoder architecture [46]. Designed to overcome the vanishing gradient problem [47]. Figure 3b and g gives the pictorial view.

#### BiGRU + CNN and GRU + CNN

BiGRU + CNN and GRU + CNN were also inspired from the encoder–decoder architecture. GRU and BiGRU act as encoders and the 1-D CNN as the decoder as shown in Fig. 3h and i.

#### XLM-Roberta (XLM-R)

XLM-R is a transformer-based, multilingual, masked language model [48], pre-trained on text in 100 languages. It delivers SOTA performance on cross-lingual classification, sequence labeling and answering questions. XLM-R_base_ was used for classification tasks,^12^ in this study.

## Experimental setup

This section addresses different combinations of hyper-parameters used by deep learning approaches for empirical investigations. Key configuration elements of the experimental platform, to train the deep learning models, were as follows: Python 3.7.9 version, NVIDIA Graphical Processing Unit (GPU) driver version 460.32.03, Compute Unified Device Architecture (CUDA) 11.2 and Tesla K80 GPU with 12 GB memory.

### Hyper-parameters for deep learning models

Design of a neural network architecture calls for tuning of diverse combinations of hyper-parameters. This ensues in optimization of the hyperparametric values, using the Grid-Search technique. The crux of any neural network is to define the right combination of parameters that yields high performance and low error rate. The following Table 4 convey the standard values of hyper-parameters, which were utilized and tuned for experimental evaluations:

**Table 4:** Range of hyper-parameter values for deep learning models

Hyper-parameters	Offensive task	Sentiment task
Learning rate	10^–5^ to 1	10^–5^ to 1
Dropout	0 to 1	0 to 1
Epochs	5 and 60 with intervals of 10	10 and 400, with an interval of 10
Word embedding dimensions	100 to 500	100 to 800
Batch size	32, 64, 128, and 256	32, 64, 128, 256 and 512
Window size	2 to 10	2 to 10
Maximum sequence length	32 and 64	8, 10, 16
Hidden units	80 to 150	80 to 150
Loss function	Cross-entropy	Cross-entropy
Activation functions	elu, selu, relu	elu, selu, relu
Optimization Algorithm	Adagrad, Adadelta, RMSprop, Stochastic Gradient Descent(SGD) and Adam	Adagrad, Adadelta, RMSprop, Stochastic Gradient Descent(SGD) and Adam

### Optimal hyper-parameter configuration

Iterative applications of the Grid-Search scheme enabled the identification of the optimal hyper-parameters that yielded SOTA weighted F1-scores, on the two code-mixed data sets. The finally selected hyper-parameters were the ones that exhibited best performance for the deep learning models. All the hyper-parameters were tuned on training and development data. On the sensible selection of optimal hyper-parameters, those parameters were exercised for evaluation on the test data. Table 5 displays a overview of the optimal hyper-parameter values for the best performing models.

**Table 5:** Overview of the optimal hyper-parameters for the best performing models for both the tasks

Task	Model	Hyper-parameters	Values
Offensive detection	GRU + CNN	Window size	4
		Max Sequence Length	64
		Epochs	50
		Batch Size	32
		Word emb dim	100
		Hidden Units	110
		Spatial Dropout	0.0228
		Dropout	0.0235
		Learning Rate	4.695e-05
		Activation Function	Selu
		Optimizer	Adam
Sentiment analysis	GRU	Window size	9
		Max Sequence Length	10
		Epochs	130
		Batch Size	256
		Word emb dim	100
		Hidden Units	110
		Dropout	0.3411
		Learning Rate	0.0001
		Optimizer	Adam

## Results and discussions

This section summarizes the results and analysis of simple deep learning models, bidirectional models, hybrid models, and transformers. Efficacy of the proposed system was evaluated in terms of the F1-score,^13^ on a held-out test data, using the Sklearn^14^ machine learning tool.

Table 6 evince the results of SOTA deep learning models for OLI in code-mixed Malayalam–English data set. The experimental evaluations show that the Recurrent Neural Network(RNN) variants, LSTM and GRU, performed better than CNNs. LSTM and GRU enhanced F1-score by marginal values of 1.15% and 1.50%, respectively, when compared with CNN, RNNs are outfitted to capture sequential information, unlike the CNNs that handle information of local context only. Bidirectional networks enable models to discern dependencies on either side of the code-mixed text, unlike unidirectional models that apprehend information only from the precedent input. Bi-directional layers can learn forward and backward features from an input sequence. Thus, bidirectional models such as BiGRU exceeded the F1-scores of unidirectional models (CNN, LSTM, GRU) by 1.55%, 0.68%, and 0.78%, respectively. Among the hybrid models, GRU + CNN and BiLSTM + CNN turned in the highest F1-score of 0.9969. Several other hybrid and bidirectional models shown in Table 4 yielded results on par with the best result.

**Table 6:** Comparison results of overall accuracy, precision, recall, and F1-score for OLI

Model	Word embedding method	Precision	Recall	F1-score	Accuracy
CNN	Word2Vec FastText	0.9901 0.9834	0.9895 0.9810	**0.9896** 0.9816	0.9895 0.9810
LSTM	Word2Vec FastText	0.9930 0.9905	0.9930 0.9900	**0.9929** 0.9901	0.9930 0.9900
GRU	Word2Vec FastText	0.9965 0.9898	0.9965 0.9890	**0.9964** 0.9891	0.9965 0.9890
BiLSTM	Word2Vec FastText	0.9937 0.9965	0.9935 0.9965	0.9935 **0.9964**	0.9935 0.9965
BiGRU	Word2Vec FastText	0.9965 0.9970	0.9965 0.9970	0.9964 **0.9969**	0.9965 0.9970
BiLSTM + CNN	Word2Vec FastText	0.9960 0.9969	0.9960 0.9970	0.9959 **0.9969**	0.9960 0.9970
BiGRU + CNN	Word2Vec FastText	0.9960 0.9852	0.9960 0.9835	**0.9959** 0.9839	0.9960 0.9835
LSTM + CNN	Word2Vec FastText	0.9950 0.9964	0.9950 0.9965	0.9949 **0.9964**	0.9950 0.9965
GRU + CNN	Word2Vec FastText	0.99701 0.9955	0.9970 0.9955	**0.9969** 0.9954	0.9970 0.9955
Transformer based classification model	XLM-R	0.9904	0.9900	0.9901	0.9900

The numbers in bold represent the highest F1-score obtained for each model

Table 7 results verify that for OLI, the proposed models performed better than the conventional models. The strength of the handcrafted transliteration and translation features cleared the way for attainment of the augmented F1-score. All the models were fine-tuned to derive optimal hyper-parameters, ensuing in a marginal outperformance by all the proposed methodologies over the published studies of contemporaneous approaches.

**Table 7:** OLD accuracy of the proposed model compared with published results in EACL 2021

System	F1-score	Precision	Recall
hate-alert [5]	0.97	0.97	0.97
SJ AJ [49]	0.96	0.96	0.96
NLP-CUET [50]	0.93	0.92	0.94
Proposed model	0.99	0.99	0.99

**Fig. 4:**
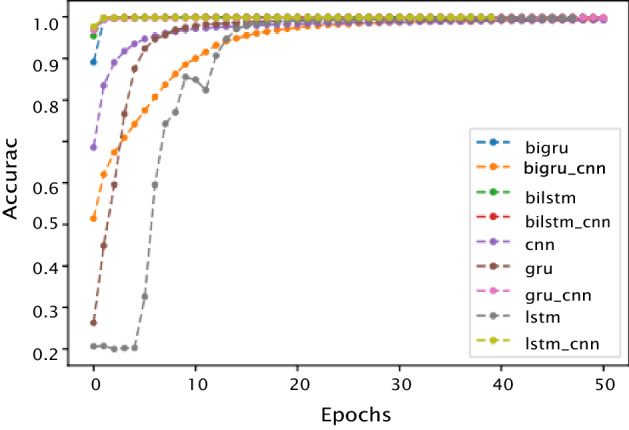
Accuracy vs epochs

**Fig. 5:**
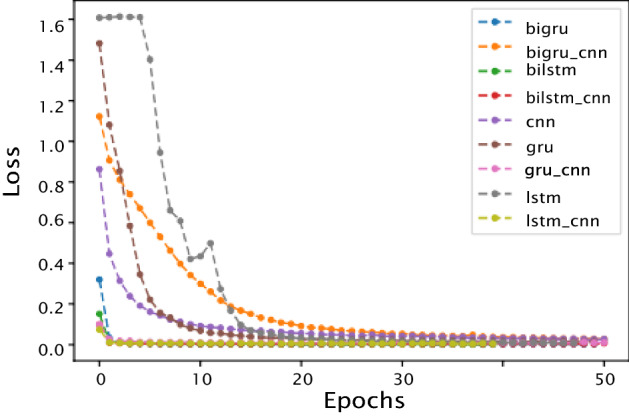
Loss vs epochs

Figures 4 and 5 present the accuracy and loss curves respectively of the training data set. Initially, the accuracy curve shows that as the number of epochs increases, the accuracy curve grows fast; at some point, all the models converge into a single line. Hence, with the rise in the number of epochs, accuracy of the models increases, reaching saturation after a few additional epochs. The loss curves, notably high at the get-go, follow steep descents after a few epochs, reaching a plateau with increased number of epochs. However, neither oscillations nor further decrease in loss was observed with additional increase in the number of epochs. As a rule, smaller the value of the loss (closer to 0) the better the performance of the deep learning models on the test data.

**Fig. 6:**
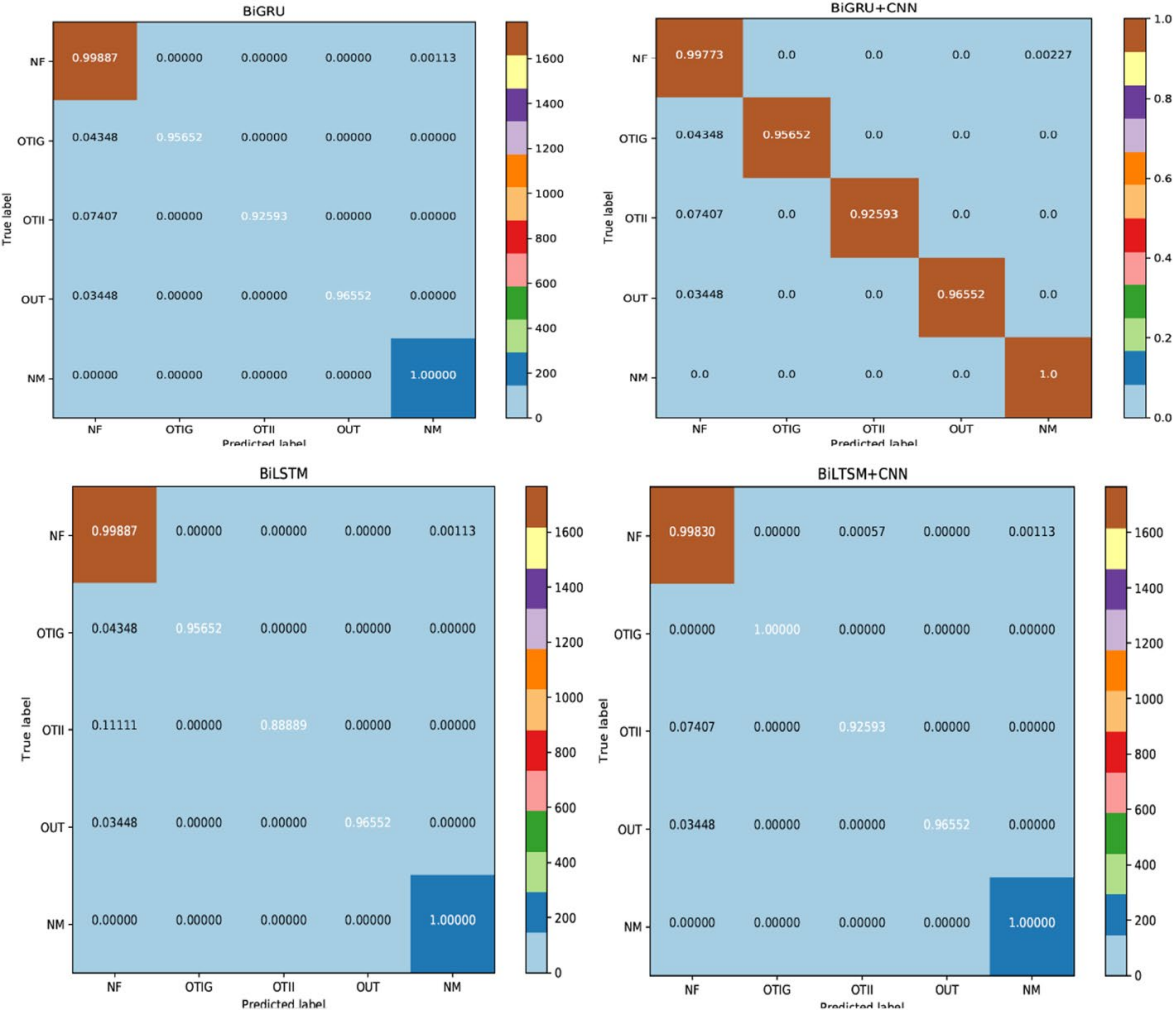

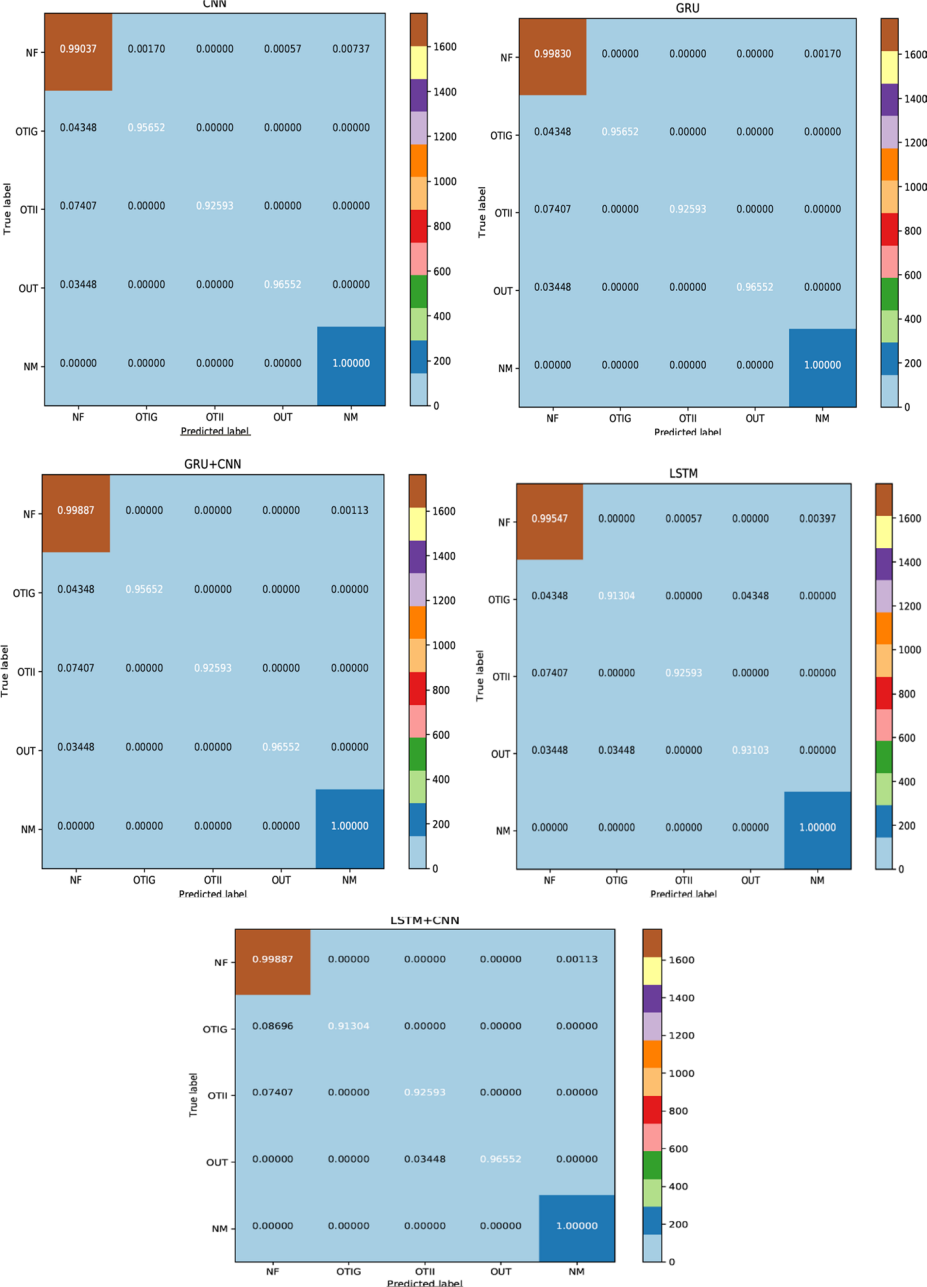
Confusion matrices of various models on offensive detection task

Albeit each model can be appraised for its accuracy, this does not furnish any insight into the aptness of this model. Figure 6 displays the Confusion Matrices which deal with the test data that validate the performance of a model. In each matrix, the x-axis represents the predicted labels, whereas the y-axis represents the veritable true labels. The diagonal elements portray the majority of normalized values, for which the predicted label and true label are equal. Matrix values in the upper and lower triangles are misclassified samples, with respect to classes in each row and column. The figure verifies the small misclassification rate for the models, which signify many correct predictions. In Table 8, the proposed methodologies of GRU + CNN, BiLSTM + CNN and BiGRU show the lowest misclassification rate of 0.003 due to high F1-score, as compared to CNN, which has the highest misclassification rate of 0.01 supervened by a low F1-score. Hence, they have a strong performance edge over CNNs. In addition, the class-wise accuracy for each of the proposed deep learning models is outstanding. Interestingly, although these proposed models rarely confuse between NM and NF, few misclassifications were observed for the other 3 classes (OTIG, OTII, OUT).

**Table 8:** Misclassification rate on offensive detection task

Model	Value
CNN	0.0104
LSTM	0.0070
GRU	0.0035
BiLSTM	0.0035
BiGRU	0.0030
BiLSTM + CNN	0.0030
BiGRU + CNN	0.0040
LSTM + CNN	0.0035
GRU + CNN	0.0030

Data summarized in Table 9 reaffirm the augmented F1-score of SA of the proposed system as being comparable to the extant benchmark criteria. F1-scores for SA, as compared to OLI tasks, are relatively low, limited by data availability. GRU gave impressive results among all the experimented models. GRU works better than CNN as the latter does not consider long term dependencies in the sentences, which is important in text analytics. GRUs are simpler in nature due to the presence of additional update and forget gates in LSTM. Unlike LSTM, GRUs are able to avoid being overfitted, as reflected in the F1-scores of the proposed deep learning models. The lowest misclassification rate of 0.236 which can be ascribed to GRUs having the highest F1-score.

**Table 9:** Comparative results for SA of overall accuracy, precision, recall, and F1-scores

Model	Word embedding method	Precision	Recall	F1-score	Accuracy
CNN	Word2Vec FastText	0.7518 0.7458	0.7455 0.7329	**0.7477** 0.7374	0.7455 0.7329
LSTM	Word2Vec FastText	0.7213 0.7392	0.6995 0.7381	0.7057 **0.7372**	0.6995 0.7381
GRU	Word2Vec FastText	0.7641 0.7607	0.7603 0.7633	0.7615 **0.7617**	0.7603 0.7633
BiLSTM	Word2Vec FastText	0.7247 0.7374	0.7225 0.7284	0.7226 **0.7297**	0.7225 0.7284
BiGRU	Word2Vec FastText	0.7020 0.7395	0.6965 0.7351	0.6961 **0.7370**	0.6965 0.7351
BiLSTM + CNN	Word2Vec FastText	0.7171 0.7380	0.6810 0.7396	0.6933 **0.7356**	0.6810 0.7396
BiGRU + CNN	Word2Vec FastText	0.7112 0.7416	0.7106 0.7203	0.7080 **0.7276**	0.7106 0.7203
LSTM + CNN	Word2Vec FastText	0.7135 0.7215	0.7151 0.7292	0.7124 **0.7225**	0.7151 0.7292
GRU + CNN	Word2Vec FastText	0.7207 0.7514	0.7121 0.7255	0.7158 **0.7336**	0.7121 0.7255
Transformer based classification model	XLM-R	0.7312	0.7299	0.7302	0.7299

The bold numbers represent the highest F1-score obtained for each model

Top 3 existing approaches versus proposed model for SA are shown in Table 10. Compared to all other systems, the proposed GRU model achieved notable improvement in F1-score, achieving a marginal 2% improvement. The key to this lies in the obligatory preprocessing stages of transliteration and translation. Supervened by the class imbalance problem, corpus of code-mixed language can be worked around, by up-sampling, to avert performance degradation.

**Table 10:** Validation of the proposed work with the published results in FIRE 2020 for SA

System	F1-score	Precision	Recall
SRJ [12]	0.74	0.74	0.74
YUN111 [12]	0.73	0.73	0.73
DT [12]	0.72	0.72	0.72
Proposed model	0.76	0.76	0.76

**Fig. 7:**
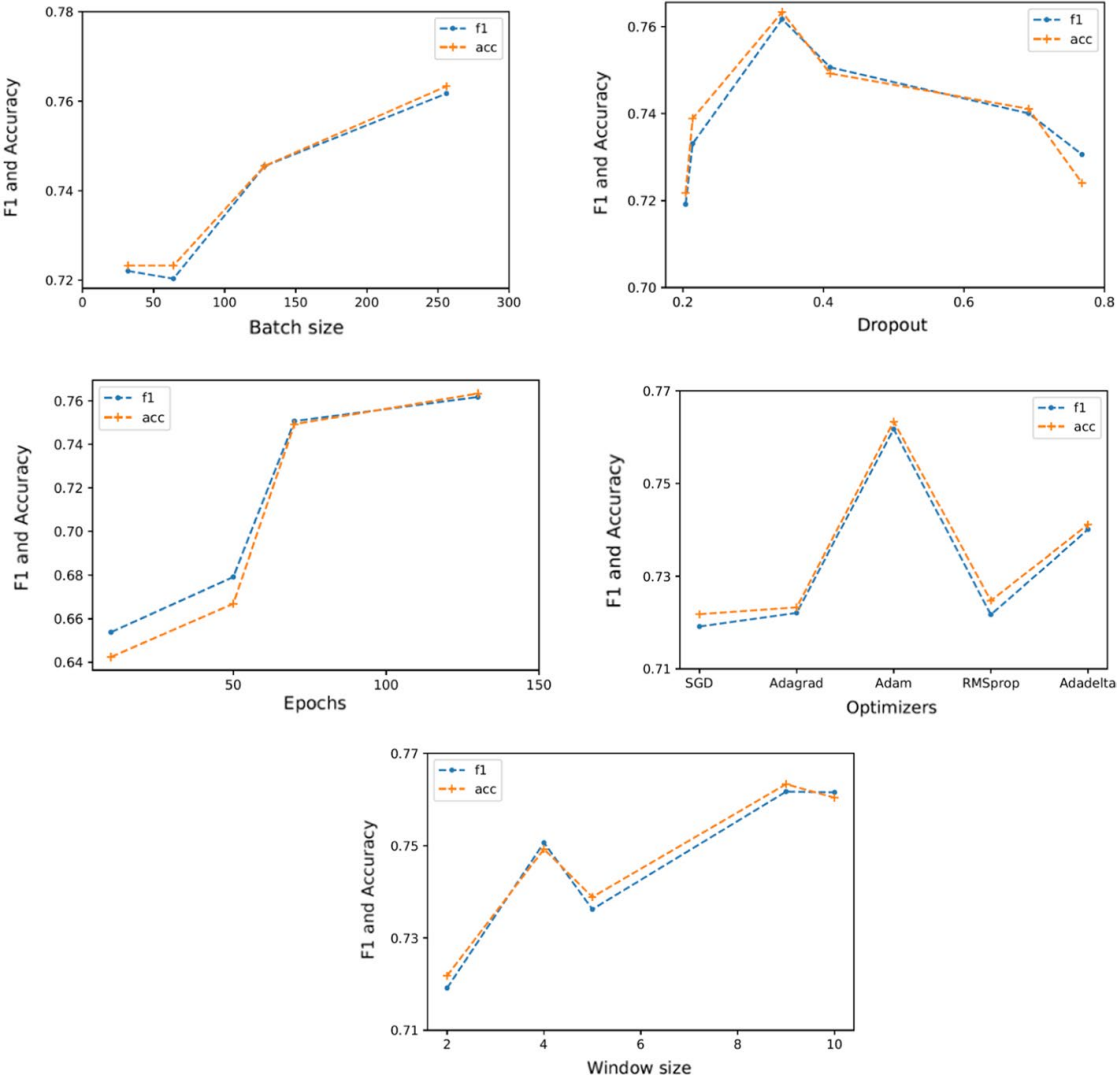
Relationship between hyper-parameters and performance metrics (F1-score (f1) and Accuracy (acc))

Figure 7 portrays how each of the five hyper-parameters are varied for the best experiment, which reportedly has the highest F1-score and accuracy for SA. As the number of epochs increases, the model is more capable of generalizing the learning. Usage of a large number of epochs ensue in overfitting problem on the training set, and the model would perform poorly for validation or test set. The maximum F1-score was attained at epoch 130. Simultaneous dropout is used as a better regularization technique, to avoid overfitting. The figure shows that the model reaches the highest performance value with a dropout value of 0.34. Next, the optimizer minimizes the loss function of the model. Among the five popular optimizers, Adam was the preferred choice as it gave the highest classification performance. Also, we have learning rate where it is responsible for the optimization of the weights. The classification performance of the model became stable when the value of the learning rate was 0.0001. Finally, the maximum performance was achieved at a window size of 9.

Table 11 presents the results of the models, with and without translation and transliteration, to draw attention to their relevance as the key preprocessing step in SA. An increase in the weighted F1-score value on the data set was seen with translation and transliteration, when compared to data set without translation and transliteration. This is a direct consequence of the rich features extracted, before translation and transliteration, from the data set of a single language corpus by the word embedding models, in contrast to a corpora of multiple languages.

**Table 11:** F1-score comparison to show the effect of translation and transliteration for sentiment analysis

Model	Without translation and transliteration	With translation and transliteration
CNN	0.6778	0.7477
GRU	0.6907	0.7617
LSTM	0.6794	0.7372
BiGRU	0.6659	0.7370
BiLSTM	0.6270	0.7297
LSTM + CNN	0.6349	0.7225
GRU + CNN	0.6663	0.7336
BiGRU + CNN	0.6780	0.7276
BiLSTM + CNN	0.6736	0.7356
Transformer based classification model(XLM-R)	0.6600	0.7302

A detailed error analysis of the proposed models, conducted to derive insightful corollaries, is shown in Table 12. As the posts in the not-Malayalam class were either in Roman or native scripts, the proposed models were able to clearly discern this class from rest of the classes. Comments are often misclassified as positive in the SA task, as majority of the data set belongs to positive class. After the positive class, Unknown state class has a greater number of examples in the code-mixed data set. Hence, unknown state has a good class-wise accuracy, after NM and positive classes. Few negative classes were mis-classified as positive which may be due to micro aggressive [51] comments posted by people. Such subtle comments complicate the analysis for researchers, in the discernment of the true nature, quantification, and automatic extraction of micro aggressions. For instance:

**Table 12:** Error analysis on predicted vs true labels for SA

Example	True label	Predicted label
Njan antarticail ninnanu malayalam ariyilla trailer adipoli	Positive	Positive
Movie ok aanu. Entertaining movie	Positive	Mixed feelings
Mara paazhu mega mairananil ninnum ethil koodutal pratheeshikaruthu 1980 kalile ra- janikanthinu padikkunnu verum chavaru mairan…	Negative	Positive
Poojappura laalunni fans dislike adichu kuru potichitund	Negative	Negative
Ithu kanditu jayasuryayude idi ormavanthu enk mathramano?	Unknown state	Unknown state
Maoist alla avnte achn vare namml adich odikkum ikkaaa	Unknown state	Positive
Adipoli but movie engane indaavuo aavo.	Mixed feelings	Mixed feelings
Lalettante stunt…. kandirunnupovum aarum… Entha oru style… Mammootty thapassirunnal polum ettante aduthu ethillaaa…	Mixed feelings	Negative
woow mammopokka ! proud of you mollywoods king ikkaa.!	Not-Malayalam	Not-Malayalam
Bahut hi acha trailer hai dil jeet liya	Not-Malayalam	Not-Malayalam

*Mara paazhu mega mairananil ninnum ethil koodutal pratheeshikaruthu 1980 kalile rajanikanthinu padikkunnu verum chavaru mairan….*

True label: Negative

Predicted label: Positive

In the above example, albeit the comments are not explicitly negative, they clearly express disapproval of the movie.

*Maoist alla avnte achn vare namml adich odikkum ikkaaa*

True label: unknown state

Predicted label: Positive

In the above example Sarcasm not detected. It was observed that the models could not capture sarcasm. It is a complex linguistic phenomena.

*Lalettante stunt…. kandirunnupovum aarum… Entha oru style. Mammootty*

*thapassirunnal polum ettante aduthu ethillaaa….*

True label : Mixed feelings

Predicted label: Negative

In above examples, system failed to predict correctly, as they did not possess the required world knowledge. Here the word thapassirunnal is a colloquial metaphor, which is used for mocking someone but the system failed to understand it.

*Movie ok aanu. Entertaining movie*

True label: Positive

Predicted label: Mixed feelings

Length of the sentences played a vital role in predicting correctly. When the length of the sentence is too short or too long the classifiers failed to predict it.

The proposed method can benefit humankind in societal perspectives. Albeit access restricted to people who know or understand English, as the Internet is rife with digital information, any small gain or acquired information must be disseminated to commoners unfamiliar or unable to access technology.

## Limitations

As the training data for SA was less than for OLD, in the code-mixed dataset, adoption of transfer learning [52] from a multilingual model would be preferable as it can further enhance the performance.

## Conclusion

This work reviewed significant research of the Malayalam–English code-mixed language, accessible in the public domain. Several deep learning models were exploited for two basic tasks: SA and OLI. The proffered method achieved impressive F1-scores, in spite of the intricacies of code-mixed language, as compared to monolingual language. Asymmetrical distribution of code-mixed data sets among different classes apropos. SA and OLD tasks, call for an up-sampling work around strategy. This research highlighted the aptness of translation and transliteration preprocessing. These major offerings coupled with data up-sampling and word embeddings, led to benchmark results for deep learning methods. As observed the proposed model achieved the best score for both the tasks. Empirical analysis of deep learning models yielded marginal improvements of 1.63% in accuracy and 1.55% in F1-score for OLD, while enhancements of 12.08% in accuracy and 9.86% in F1-score for SA were turned in. The readers are anticipated to make informed choices in their selection of the deep learning model, or perhaps, a combination of models that may be a judicious choice.

## Future work

Extant work of code-mixed languages can be broadened to handle more than two languages, for multilingual societies. Despite advancements reported in the code-mixed domain, limited availability of sentimental analysis data, call for improvement in a model’s accuracy [F1-scores]. Data augmentation by selective addition of class-specific data is expected to lower the misclassification rate. On the design section, ensemble approach should be probed to ascertain its relevance and efficacy.

## Data Availability

Not applicable. For any collaboration, please contact the authors.

## Footnotes

1 https://en.wikipedia.org/wiki/History_of_the_British_Raj.

2 https://www.britannica.com/topic/Dravidian-languages.

3 https://en.wikipedia.org/wiki/Vatteluttu.

4 https://en.wikipedia.org/wiki/Abugida.

5 https://dravidian-codemix.github.io/2020/.

6 https://competitions.codalab.org/competitions/27654.

7 https://www.nltk.org/.

8 https://pypi.org/project/google-trans-new/.

9 https://www.kaggle.com/pierremegret/gensim-word2vec-tutorial.

10 https://pypi.org/project/numpy/.

11 https://keras.io/api/layers/corelayers/embedding/.

12 https://huggingface.co/transformers/.

13 https://pypi.org/project/scikit-learn/.

14 https://scikit-learn.org/.
